# Multiple Functions and Mechanisms Underlying the Role of METTL3 in Human Cancers

**DOI:** 10.3389/fonc.2019.01403

**Published:** 2019-12-12

**Authors:** Wenhui Zheng, Xiaoshen Dong, Yan Zhao, Shuo Wang, Haiyang Jiang, Mingdi Zhang, Xinyu Zheng, Ming Gu

**Affiliations:** ^1^Department of Anesthesiology, The Shengjing Hospital of China Medical University, Shenyang, China; ^2^Department of Breast Surgery, The First Affiliated Hospital of China Medical University, Shenyang, China; ^3^Department of Breast Surgery, Obstetrics and Gynecology Hospital of Fudan University, Shanghai, China; ^4^Lab 1, Cancer Institute, The First Affiliated Hospital of China Medical University, Shenyang, China

**Keywords:** METTL3, m^6^A, cancer, mechanism, pathway

## Abstract

Methyltransferase-like 3 (METTL3), a predominantly catalytic enzyme in the N^6^-methyladenosine (m^6^A) methyltransferase system, is dysregulated and plays a dual role (oncogene or tumor suppressor) in different human cancers. The expression and pro- or anticancer role of METTL3 in different cancers remain controversial. METTL3 is implicated in many aspects of tumor progression, including tumorigenesis, proliferation, invasion, migration, cell cycle, differentiation, and viability. Most underlying mechanisms involve multiple signaling pathways that rely on m^6^A-dependent modification. However, METTL3 can also modulate the cancer process by directly promoting the translation of oncogenes via interaction with the translation initiation machinery through recruitment of eukaryotic translation initiation factor 3 subunit h (eIF3h). In this review, we summarized the current evidence on METTL3 in diverse human malignancies and its potential as a prognostic/ therapeutic target.

## Introduction

Chemical modifications of nucleobases are critical for controlling gene expression at different levels, which subsequently induces changes in protein translation and modulates signaling pathways. N^6^-methyladenosine (m^6^A) modification of various RNAs, including eukaryotic messenger RNAs (mRNAs) (1), microRNAs (miRNAs) (2), and long non-coding RNAs (lncRNAs) (3) is enriched in near stop codon and 3′ untranslated terminal region (UTR) (4) and translated near 5′ UTR in a cap-independent manner (5) and has been considered one of the most ubiquitous, reversible and abundant internal modifications on RNA molecules.

**Figure 1:**
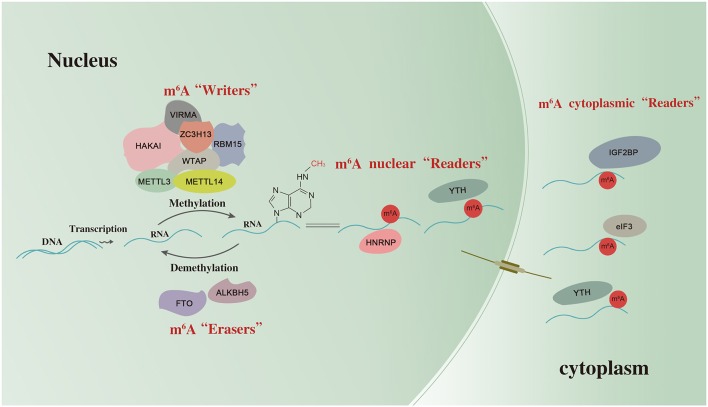
The scheme of m^6^A RNA methylation. m^6^A RNA methylation is regulated by “writers”, “erasers,” and “readers”. METTL3, methyltransferase-like 3; METTL14, methyltransferase like 14; WTAP, Wilms tumor 1-associated protein; RBM15, RNA binding motif protein 15; ZC3H13, zinc finger CCCH-type containing 13; VIRMA, vir like m6A methyltransferase associated; FTO, the fat mass and obesity-associated protein; ALKBH5, alkB homolog 5; YTH, YT521-B homology domain-containing protein; eIF3, eukaryotic translation initiation factor 3; IGF2BP, IGF2 mRNA binding proteins; HNRNP, heterogeneous nuclear ribonucleoprotein.

In recent years, substantial progress has been made in understanding m^6^A modifications in various metabolic and infectious diseases, as well as cancer (6). In addition, a variety of studies have shown that m^6^A modification plays promotive or inhibitory roles in various cancers (7). Upon alteration of m^6^A regulatory genes or a change in the expression of proteins related to m^6^A methylation, m^6^A impacts the initiation and progression of various human malignancies through diverse mechanisms and is involved in different biological processes, including viral infection (8), immune responses, tissue renewal (8–10), stem cell differentiation, and motility (11), and therefore exerts a profound impact on cancer development (8).

m^6^A deposition is accomplished by a methyltransferase (MTase) complex referred to as “writers.” In mammalian cells, m^6^A MTase activity on mRNA requires at least two separate proteins: MT-A (200 KDa) and MT-B (800 KDa). MT-A is a multimeric protein that contains a 70-KDa S-adenosylmethionine-binding subunit referred to as MT-A70, which is also known as METTL3 (12). METTL3 is a predominantly catalytic enzyme of m^6^A MTase systems and belongs to the class I MTase family. It usually forms a stable heterodimeric complex with METTL14 (13). Along with the m^6^A-METTL associated complex composed of Wilms tumor 1-associated protein (WTAP), RNA binding motif protein 15 (RBM15), zinc finger CCCH-type containing 13 (ZC3H13), vir like m6A MTase associated (VIRMA), and HAKAI, they catalyze the formation of m^6^A. The fat mass and obesity-associated protein (FTO) and alkB homolog 5 (ALKBH5) are categorized as “erasers” which selectively remove the methyl code from target RNAs and reverse the methylation. YT521-B homology (YTH) domain-containing protein, eukaryotic translation initiation factor 3 (eIF3), IGF2 mRNA binding proteins (IGF2BP) families, and heterogeneous nuclear ribonucleoprotein (HNRNP) protein families are categorized as “readers” which decode m^6^A methylation and generate a functional signal (6, 14). Thus, m^6^A may affect RNAs splicing, stability, transcription, and translation (15) as shown in Figure 1.

METTL3 plays a pivotal role in all stages of the RNA life cycle involving m^6^A, including pre-mRNA splicing (16), nuclear export (17), translation regulation (18), mRNA decay (19), and miRNA processing (20). With the help of other components of “writers,” the METTL3 catalytic process modifies most m^6^A sites and has been considered the most common m^6^A pathway, especially in mRNA (21). METTL3 has been demonstrated to be dysregulated in diverse human malignancies and involved in many aspects of carcinogenesis. Zhou et al. showed that METTL3 and METTL14 exhibited higher expression in clear cell renal cell carcinoma than in normal samples and that patients with deletion of METTL3 had poorer overall survival (OS) and disease-free survival (DFS) (22). Liu et al. revealed that the proliferation, viability, and migration of gastric cancer cells with METTL3 silencing *in vitro* were significantly inhibited compared with those of control cells (23). This research group also noted that METTL3 expression was associated with biological processes, including adipogenesis, the mTOR pathway, and reactive oxygen species (24). Taketo et al. found that METTL3-deleted cells showed higher sensitivity to anticancer reagents, indicating that METTL3 may promote drug resistance in pancreatic cancer (25). The abovementioned study suggested that METTL3 plays a role in oncogenesis. High expression of METTL3 may predict poor survival and drug resistance in patients.

In contrast, Deng et al. showed that high expression of METTL3 was significantly associated with longer survival time and METTL3 played a tumor-suppressive role in colorectal cancer (26).

The role of METTL3 in cancer cells is controversial (27). The contradictory conclusions reached in previous studies must be related to the differences in the mechanisms of origin of different cancers. In this review, we summarized the recent advances made in relation to METTL3 dysregulation and its dual role coupled with the underlying mechanisms in various human cancers.

## METTL3 Dysregulation in Human Cancers

In the majority of cancer research, METTL3 has been found to be upregulated and to play an oncogenic role accompanied by increased m^6^A levels compared with those in normal tissues or cell lines. However, some pieces of research have yielded opposite results even in the same cancer type, as shown in Table 1.

**Table 1:** Expression, clinical significance, and biological function of METTL3 in various cancers.

**Cancer type**	**Expression**	**Role**	**Biological function**	**References**
Bladder cancer	Upregulated	Oncogene	Proliferation, invasion, tumorigenesis	(28–30)
	/	Tumor suppressor	Proliferation	(31)
Renal cell carcinoma	Downregulated	Tumor suppressor	Proliferation, migration, invasion, cell cycle	(32)
	Upregulated	Tumor suppressor	Proliferation, migration	(22)
Lung cancer	Upregulated	Oncogene	Proliferation, invasion, migration	(33–36)
Colorectal cancer	Upregulated	Oncogene	Stem cell differentiation, migration, tumorigenesis	(34, 37, 38)
	/	Tumor suppressor	Proliferation, migration, invasion	(26)
Glioma	Upregulated	Oncogene	Proliferation	(39)
	/	Tumor suppressor	Proliferation, stem cell differentiation	(40)
Breast cancer	Upregulated	Oncogene	Proliferation, apoptosis, migration	(41, 42)
	Downregulated	Tumor suppressor	Cell viability and colony formation	(43)
Leukaemic	Upregulated	Oncogene	Proliferation, cell cycle, tumorigenesis, stem cell differentiation	(44, 45)
Osteosarcoma	Upregulated	Oncogene	Proliferation, migration, invasion	(46)
Gastric cancer	Upregulated	Oncogene	Proliferation, migration, invasion	(23, 47, 48)
Melanoma	Upregulated	Oncogene	Colony formation, invasion	(49)
Ovarian carcinoma	Upregulated	Oncogene	Proliferation, motility, invasion, tumorigenesis	(50)
Hepatocellular carcinoma	Upregulated	Oncogene	Proliferation, migration, colony formation, tumorigenesis	(51)
Pancreatic cancer	/	Oncogene	Chemoresistance	(25)

*METTL3, methyltransferase like 3*.

### METTL3 in Urological Tumors

Recent studies noted that METTL3 was drastically upregulated in bladder cancer tissues and was related to tumor histological grade. Patients with high expression of METTL3 had poor prognosis and reduced survival time (28–30). Knockdown of *METTL3* significantly reduced bladder cancer cell invasion, proliferation, and survival *in vitro* and tumorigenicity *in vivo* (31). The above studies proved that METTL3 acts as an oncogene in bladder cancer.

However, Zhao et al. showed that deletion of *METTL3* significantly increased the proliferation of bladder cancer cell line 5637. Wild-type *METTL3* successfully restored the normal growth rate and somatic mutations in *METTL3* may disrupt the m^6^A methylation process and promote cancer cell growth. METTL3 acts as a tumor suppressor gene in bladder cancer (31). Similarly, Li et al. showed that METTL3 expression was lower in renal cell carcinoma samples compared with adjacent non-tumor samples. Negative METTL3 expression was significantly associated with larger tumor sizes and higher histological grade. Patients with high METTL3 expression had obviously extended survival time. Moreover, knockdown of *METTL3* appreciably promoted cell proliferation, migration, and invasion and induced G0/G1 arrest, suggesting that METTL3 may act as a tumor suppressor in renal cell carcinoma (32).

### METTL3 in Lung Cancer

METTL3 was upregulated in primary human lung adenocarcinomas compared with adjacent normal tissues, and METTL3 depletion suppressed the growth of lung cancer xenografts *in vivo* (33, 34). In addition, Du et al. revealed that METTL3 expression was higher in non-small cell lung carcinoma tissues than in adjacent tissues (35). METTL3 promotes the proliferation, survival, migration, and invasion of human lung cancer cells (34, 36). Collectively, these studies on METTL3 in lung cancer suggest the oncogenic role of METTL3.

### METTL3 in Colorectal Cancer

Liu and colleagues compared the m^6^A-related genes in colorectal cancer and found that most m^6^A-related genes, including *METTL3*, were upregulated, except *METTL14, YTHDF3*, and *ALKBH5* (37). METTL3 expression was consistently elevated in recurrent colorectal cancer, matched lymph node, and metastatic liver tissues. Colorectal cancer patients with high METTL3 expression had reduced OS and DFS times (37, 38). Knockdown of *METTL3* in colorectal cancer cells significantly inhibited tumorigenesis and metastasis, cell self-renewal, and the frequency and migration of stem cells *in vitro* and *in vivo* (34), suggesting the oncogenic role of METTL3 in colorectal cancer.

However, Deng et al. noted that positive expression of METTL3 inhibits cell proliferation, migration, and invasion in colorectal cancer (26). Negative expression of METTL3 was significantly correlated with larger tumor size and metastasis. Multivariate analysis indicated that METTL3 expression status is an independent prognostic factor for DFS (26). In addition, knockdown of *METTL3* promoted tumor proliferation and metastasis. The tumor suppressor role of METTL3 in this research was related to the P38/ERK pathway (26).

### METTL3 in Glioma

METTL3 expression was found to be elevated in glioma stem-like cells and attenuated during differentiation (39). Glioblastoma tumors exhibited elevated levels of METTL3 transcripts, and silencing METTL3 inhibited tumor growth coupled with prolonged survival of mice *in vivo* (39), suggesting the oncogenic role of METTL3 in glioblastoma. However, in another study on the function of m^6^A in glioblastoma, METTL3 overexpression inhibited stem cell growth and self-renewal, accompanied by suppressed tumor progression (40).

### METTL3 in Breast Cancer

Recent studies showed that METTL3 was upregulated in breast cancer tissue and cells. Knockdown of METTL3 reduced cell proliferation and accelerated apoptosis and migration by targeting Bcl-2, suggesting the oncogenic role of METTL3 in breast cancer (41, 42). However, another study that explored key m^6^A-related enzymes via combined analysis of data from the ONCOMINE and The Cancer Genome Atlas databases with 36 pairs of breast cancer and adjacent non-cancerous tissues, revealed that the expression of all m^6^A methylases, including METTL3, was reduced in breast cancer. These researchers also noted that METTL3 and METTL14 were upregulated in the normal breast-like and luminal breast cancer subtypes compared with the basal-like and HER2-overexpressing types. MTase overexpression induced m^6^A expression and inhibited tumor cell viability and colony formation. Thus, METTL3 acts as a tumor suppressor (43).

### METTL3 in Leukemia

Vu et al. showed that METTL3 mRNA and protein are expressed more abundantly in acute myeloid leukemia cells than in healthy human hematopoietic stem/progenitor cells or other types of tumor cells. Knockdown of *METTL3* promoted cell differentiation accompanied by reduced cell proliferation (44). Moreover, another study revealed that knockdown of *METTL3* results in differentiation of leukemic cells, failure to establish leukemia in immunodeficient mice, coupled with cell cycle arrest (45). Collectively, research in leukemia suggests that METTL3 is upregulated and significantly related to tumor cell differentiation, tumor formation, the cell cycle, and proliferation.

### METTL3 in Other Cancers

METTL3 and m^6^A were upregulated in human osteosarcoma (46), gastric cancer (23, 47, 48), melanoma (49), ovarian carcinoma (50), and hepatocellular carcinoma (51). Its expression level gradually increased with the increasing tumor stage and grade. METTL3 was an indicator of poor prognosis and *METTL3* silencing inhibited the proliferation, migration, and invasion abilities of cells, colony formation, and motility (24, 47–52), demonstrating that METTL3 plays an oncogenic role in these cancers. Taketo et al. reported that a METTL3-knockdown pancreatic cancer cell line showed higher sensitivity to anticancer agents such as gemcitabine, 5-fluorouracil, cisplatin, and irradiation, suggesting that METTL3 is a potential target for the enhancement of therapeutic efficacy (25).

## Regulatory Mechanisms of METTL3

**Figure 2:**
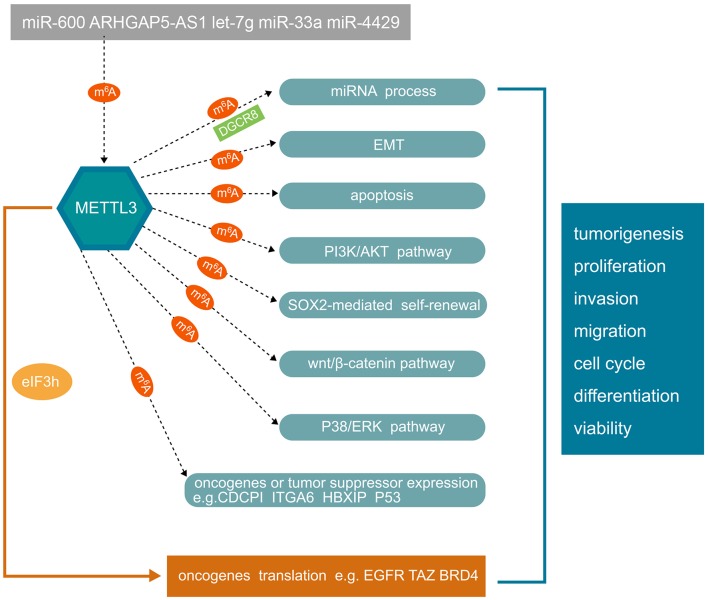
The mechanisms of METTL3 involved in human cancer progression. Non-coding RNAs can regulate METTL3 expression. Conversely, METTL3 can regulate miRNA process in m^6^A manner. METTL3 plays an important role in tumorigenesis, tumor cell proliferation, invasion, migration, cell cycle, stem cell differentiation, and viability in an m^6^A-dependent manner. The underlying mechanisms involve EMT, apoptosis, PI3K/AKT, SOX2-mediated self-renewal, wnt/β-catenin, and P38/ERK pathways. In addition, METTL3 can also regulate the expression of oncogenes or tumor suppressors in an m^6^A-dependent manner. METTL3 can also directly regulate oncogene translation independently of m^6^A by recruiting eIF3h. METTL3, methyltransferase-like 3; EMT, epithelial-mesenchymal transition; PI3K, phosphatidylinositol 3-kinase; SOX2, SRY-box transcription factor 2; ERK, extracellular regulated MAP kinase; CDCP1, CUB domain containing protein 1; ITGA6, integrin subunit alpha 6; HBXIP, mammalian hepatitis B X-interacting protein; EGFR, epidermal growth factor receptor; BRD4, bromodomain containing 4.

As mentioned above, METTL3 is implicated in many aspects of human cancer cell progression, which has prompted many researchers to explore its possible molecular mechanism. Next, we summarize the results of studies on the main pathways of METTL3 in various cancers as shown in Figure 2.

### METTL3 in the Downstream of Non-coding RNAs

miRNAs or lncRNAs can modulate the expression of oncogenes or tumor suppressors by targeting the 3′UTR of METTL3 in an m^6^A-dependent manner. Wei et al. showed that miR-600 can reverse the effect of METTL3 overexpression, as well as overexpression of the related genes in the PI3K/AKT and β-catenin/stat3 signaling pathways (36). The lncRNA ARHGAP5-AS1 can stimulate m^6^A modification of ARHGAP5 mRNA to stabilize ARHGAP5 mRNA, which is upregulated in gastric cancer, in the cytoplasm to promote chemoresistance by recruiting METTL3 (52). Hepatitis B X-interacting protein (HBXIP) upregulates METTL3 in breast cancer cells by inhibiting the miRNA let-7g, which downregulates the expression of METTL3 by targeting its 3′UTR. Conversely, METTL3 promotes the expression of HBXIP through m^6^A modification (42). Du et al. showed that miR-33a can attenuate non-small cell lung cancer cell proliferation by targeting the 3′UTR of METTL3 mRNA (35). Moreover, miR4429 inhibits gastric cancer progression by targeting METTL3 to hinder m^6^A-induced stabilization of SEC62 (53).

### METTL3 Modulates miRNA Processing via DGCR8

Alarcon et al. found that METTL3 promotes the maturation of miRNAs by interacting with the microprocessor protein DGCR8 (2, 20). Han et al. found that METTL3 positively modulates pri-miR221/222 processing in an m^6^A-dependent manner by interacting with DGCR8, resulting in a reduction in the PTEN level, which ultimately results in proliferation of bladder cancer (29). Wang et al. explored the function and mechanism of METTL3 and m^6^A in colistin-induced kidney injury and found that METTL3 interacts with DGCR8 and positively modulates the processing of mature miR-873-5p in an m^6^A-dependent manner (54). Collectively, this evidence suggests that DGCR8 plays an important role in the m^6^A-dependent regulation of miRNA processing by METTL3. METTL3 can modulate the expression of oncogenes or tumor suppressors by influencing miRNA maturation and processing via interaction with DGCR8.

### METTL3 Regulates the Epithelial-Mesenchymal Transition Pathway

The ability of epithelial cells to undergo transition to a mesenchymal phenotype during malignant progression, termed epithelial-mesenchymal transition (EMT), is now widely accepted as a core biological process (55). Liu et al. demonstrated that after knockdown of METTL3 in gastric cancer cells, the level of α-smooth muscle actin (α-SMA) was significantly reduced, while the expressions of the mesenchymal markers N-cadherin and vimentin were not markedly changed, suggesting that METTL3 silencing partially impairs EMT progression in gastric cancer cells (23). Yue et al. showed that METTL3 mediated m^6^A modification of zinc finger MYM-type containing 1 (ZMYM1) which mediated the repression of E-cadherin promoter and facilitated EMT and metastasis in gastric cancer (48). Relying on the reader ELAVL1, Lin et al. showed that m^6^A modification regulates EMT and that knockdown of *METTL3* impairs migration, invasion and EMT both *in vivo* and *in vitro* (56). METTL3 overexpression promoted the accumulation of MMP2 and N-cadherin in melanoma cells (49). Hua et al. showed that METTL3 promotes EMT by upregulating the receptor tyrosine kinase AXL in ovarian carcinoma (50). The above mentioned research suggests that METTL3 plays an oncogenic role by promoting the EMT process.

### METTL3 Regulates Apoptosis

METTL3 inhibition can induce cancer cell apoptosis by regulating the expression of apoptosis-related genes in an m^6^A-dependent manner. Lin et al. showed that downregulation of METTL3 increases the levels of the positive regulators Bax and active caspase-3 but decreases the expression of Bcl2, a negative regulator of apoptosis, suggesting that downregulation of METTL3 activates this apoptosis-related pathway (47). Wei et al. showed that nuclear METTL3 increases the Bax/Bcl2 ratio in lung cancer cells and that METTL3 knockdown strongly increases the levels of cleaved caspase3 and PARP, implying that knockdown of METTL3 induces the mitochondrial apoptotic pathways in lung cancer cells (36).

### METTL3 and the PI3K/AKT Pathway

Knockdown of METTL3 results in a reduction in m^6^A and subsequently promotes cancer cell proliferation and invasion by activating PI3K-AKT signaling. Several studies noted that knockdown of METTL3 inhibited PI3K expression and reduced the levels of the phosphorylated form of AKT, ribosomal protein S6 kinase B1 (p70S6K, an AKT downstream effector), β-catenin, and cyclin D1 (36, 44, 47, 57). METTL3 knockdown significantly suppressed cell proliferation, and METTL3 acted as an oncogene in acute myeloid leukemia cells by suppressing Myc expression via a reduction in the m^6^A level in Myc mRNA (44, 58). Also, Cheng et al. revealed that the major role of m^6^A modification in hematopoietic stem cell differentiation arises from its ability to regulate symmetric commitment by controlling Myc mRNA stability (59).

**Figure 3:**
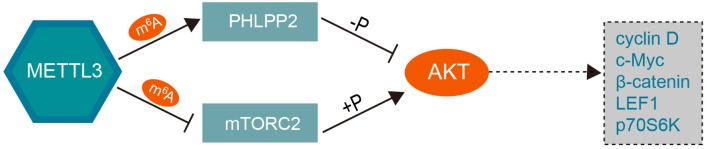
The mechanism underlying the role of METTL3 in the PI3K/AKT pathway. Overexpression of METTL3 coupled with increased m^6^A level promotes the negative AKT regulator PHLPP2 and inhibits the expression of the positive AKT regulator mTORC2, which subsequently decrease the AKT phosphorylation level and downstream effector. Knockdown of *METTL3* can activate the PI3K/AKT pathway by positively regulating the phosphorylation of AKT. METTL3, methyltransferase-like 3; PHLPP2, PH domain leucine-rich-repeat protein phosphatase 2; LEF1, lymphoid enhancer binding factor 1; p70S6K, ribosomal protein S6 kinase B1.

In addition, PH domain leucine-rich-repeat protein phosphatase 2 (PHLPP2), a tumor suppressor that inhibits cancer cell proliferation and invasion, has been proven to be a negative regulator of AKT (60). METTL3 can promote miR-25 maturation and subsequently target PHLPP2 and AKT (61). Further, Liu et al. showed that decreased expression of METTL3 results in a reduction in m^6^A methylation, which, in turn, leads to decreased expression of PHLPP2 and increased expression of the positive AKT regulator mTORC2 (62). These findings revealed that knockdown of METTL3 can activate the PI3K-AKT pathway by promoting AKT phosphorylation via modulation of the expression of AKT regulators such as PHLPP2 and mTORC2 in an m^6^A-dependent manner, as shown in Figure 3.

### METTL3 and SOX2-Mediated Stem Cell Differentiation

Sex-determining region Y (SRY)-box transcription factor 2 (SOX2) is a transcription factor whose activity is associated with cancer stem cell differentiation. High SOX2 levels are usually associated with poor outcomes. SOX2 is an important marker for the promotion of tumor initiation and proliferation and participates in tumor metastasis (63–65). Li and her colleagues found that after knockdown of METTL3 in colorectal cancer cells, the genes with the greatest expression changes were enriched in the stem cell differentiation pathway. Among these genes, *SOX2* exhibited the most consistently decreased m^6^A level in METTL3-knockdown colorectal cancer cells compared with control cells (39). Li et al. showed that the m^6^A reader IGF2BP2 bound to the SOX2 coding sequence (CDS) region to enhance SOX2 mRNA stability in an m^6^A-dependent manner (38). After METTL3 inhibition, the expression levels of cancer stem cell surface antigens such as CD133, CD44, and epithelial cell adhesion molecule were markedly reduced. In addition, decreased sphere numbers and sizes as well as a markedly reduced frequency of stem cells were observed. Moreover, the expressions of downstream *SOX2* genes, including those of cyclin D1 and Myc, were consistently suppressed. Exogenous overexpression of a *SOX2* mutant without the 3′UTR reversed the inhibitory effect of neurosphere formation in METTL3-knockdown glioma stem-like cells (39). Collectively, the research on METTL3 and SOX2 suggests that METTL3-mediated m^6^A modification regulates SOX2-associated stem cell self-renewal and tumor progression.

### Other m^6^A-Dependent Mechanisms

METTL3 can regulate tumor cell progression by modulating the expression of oncogenes such as CUB domain-containing protein 1 (CDCP1) (30), integrin subunit alpha 6 (ITGA6) (66), and mammalian HBXIP (42) or tumor suppressors [such as P53 (67)] in an m^6^A-dependent manner. Miao et al. demonstrated that METTL3 silencing decreased the expression of lymphoid enhancer-binding factor 1 (LEF1) because knockdown of METTL3 reduced the m^6^A level and shortened the half-life of LEF1 mRNA transcripts and subsequently inhibited the wnt/β-catenin pathway in human osteosarcoma (46). Deng et al. showed that in colorectal cancer cells, knockdown of METTL3 promotes cell proliferation and migration via activation of p-p38 and p-ERK, possibly indicating that METTL3 inhibits colorectal cancer cell proliferation and migration by modulating the P38/ERK pathway (26). Wang et al. showed that METTL3-mediated m^6^A modification of HDGF promotes tumor angiogenesis and that there was a correlation between nuclear HDGF level and glycolysis in gastric cancer cells, both of which were correlated with subsequent tumor growth and liver metastasis (68).

### m^6^A-Independent Mechanisms

METTL3 itself participates in controlling the translation of some m^6^A-containing mRNAs, such as the epidermal growth factor receptor, and the expression of the Hippo pathway effector TAZ, thus affecting Myc and RAS levels in lung cancer independently of m^6^A reader proteins (34). Choe et al. showed that the METTL3-eIF3h complex enhances the translation of bromodomain containing 4 (BRD4), which is also modified by m^6^A in lung cancer cells when tethered to reporter mRNA at sites near the stop codon, supporting an mRNA looping mechanism for ribosome recycling and translational control (33, 69). METTL3 can promote cancer cell growth, survival, and invasion by recruiting eIF3h to the translation initiation complex and directly promotes oncogene translation independently of its MTase activity (34).

## Discussion and Outlook

The data described in this review suggest that METTL3 is upregulated in most cancer tissues and cell lines and plays an oncogenic role in tumor formation and progression (29). However, other data suggest converse conclusions about the expression and role of METTL3 in bladder cancer, renal cell carcinoma, colorectal cancer, glioma, and breast cancer. The contradictory expression patterns and functions of METTL3 may be largely attributed to differences in the tumor tissue origin, extracellular microenvironment, upstream and downstream regulatory factors, and research methods.

As noted herein, m^6^A modification plays a dual and important role in human cancer progression (70). METTL3 is a predominant MTase for m^6^A modification, and its underlying mechanism must be complex, involving multiple molecules and pathways. The lncRNA ARHGAP5-AS1, miR-600, miRNA let-7g, and miR-33a can influence human cancer progression by targeting METTL3 (35, 36, 52, 53). Conversely, METTL3 can promote pri-miRNA processing by interacting with DGCR8 (2, 20, 54). Dysregulation of METTL3 in various cancers can also influence cancer cell EMT (23, 49, 50), apoptosis (36, 47), and stem cell self-renewal (38, 39), which have been identified to be important in cancer progression. In addition, METTL3-mediated m^6^A modification can directly regulate the transcription and translation of oncogenes and tumor suppressors coupled to the most of the important pathways involved in cancer cell progression, such as the PI3K/AKT (36, 44, 47, 57, 60, 62), wnt/β-catenin (46), and P38/ERK (26) pathways. METTL3 can also regulate cancer-related gene expression via m^6^A modification (30, 42, 66, 67). In conclusion, METTL3-related m^6^A regulatory genes involve multiple pathways and the opposing role of METTL3 in different cancer types may be associated with genes with opposing function, some of which we currently do not know.

In addition, METTL3 can promote tumor progression by regulating oncogene translation independently of m^6^A reader proteins and its MTase activity (34). METTL3 is tethered to reporter mRNA at sites near the stop codon by recruiting eIF3h (33, 34, 69). This observation may help to explain the controversy regarding the expression and role of METTL3 in cancer cells.

The role of m^6^A in disease occurrence and development has received more and more attention in recent years. Especially in the prevention and treatment of malignancies, m^6^A and its related factors are expected to become new prognostic indicators and therapeutic targets. The research pertaining to m^6^A-related enzyme inhibitors has focused mainly on “erasers” (71–73), although it is still at the preliminary stage.

As mentioned above, METTL3 appears to be a predominantly catalytic enzyme in the m^6^A process. The research about the role METTL3 in various cancers revealed that METTL3 has great potential for clinical application by serving as a new diagnostic/prognostic/treatment target. The mechanism underlying the effects of METTL3 is complex and implicated in multiple signaling pathways. The precise molecular mechanisms underlying the role of METTL3 in cancer initiation and progression are not thoroughly understood and require further systematic investigation. Further studies are needed to overcome the challenges in gaining a comprehensive understanding of the potential and limitations of METTL3 and m^6^A in cancer diagnosis and treatment.

## Conclusion

METTL3 is dysregulated and plays a dual role in various types of human cancers. Through m^6^A modification, METTL3 modulates tumor cell proliferation, invasion, migration, tumor formation, and drug resistance. These effects are orchestrated through multiple pathways, such as the miRNA processing, EMT, apoptosis, stem cell self-renewal, and PI3K/AKT pathways. Non-coding RNAs can upregulate or downregulate METTL3 expression. In addition, METTL3 can promote oncogene translation independently of m^6^A readers by recruiting eIF3h. METTL3 has great potential for clinical application by serving as a new diagnostic/prognostic/treatment target. However, further studies are still needed to clarify the exact details of METTL3 expression, roles, and mechanisms in human cancers.

## Author Contributions

WZ and MG searched PubMed about METTL3 and m^6^A in human cancers and wrote the draft. XZ and MZ summarized the dyregulation and different functions in various human cancers. YZ and HJ searched the Kegg pathway and classified the complex mechanisms. XD and SW drawed the figures attached.

### Conflict of Interest

The authors declare that the research was conducted in the absence of any commercial or financial relationships that could be construed as a potential conflict of interest.
